# Introducing Obliquely Perforated Phononic Plates for Enhanced Bandgap Efficiency

**DOI:** 10.3390/ma11081309

**Published:** 2018-07-28

**Authors:** Saeid Hedayatrasa, Mathias Kersemans, Kazem Abhary, Wim Van Paepegem

**Affiliations:** 1 Department of Materials, Textiles and Chemical Engineering, Ghent University, Technologiepark-Zwijnaarde 903, 9052 Zwijnaarde, Belgium; mathias.kersemans@ugent.be (M.K.); wim.vanpaepegem@ugent.be (W.V.P.); 2 School of Engineering, University of South Australia, Mawson Lakes, SA 5095, Australia; kazem.abhary@unisa.edu.au

**Keywords:** Phononic Crystal, Plate, Oblique Perforation, Bandgap

## Abstract

Porous phononic crystal plates (PhPs) that are produced by perpendicular perforation of a uniform plate have well-known characteristics in selective manipulation (filtration, resonation, and steering) of guided wave modes. This paper introduces novel designs of porous PhPs made by an oblique perforation angle. Such obliquely perforated PhPs (OPhPs) have a non-uniform through-the-thickness cross section, which strongly affects their interaction with various wave mode types and therefore their corresponding phononic properties. Modal band analysis is performed in unit-cell scale and variation of phononic bandgaps with respect to the perforation angle is studied within the first 10 modal branches. Unit-cells with arbitrary perforation profile as well as unit-cells with optimized topology for maximized bandgap of fundamental modes are investigated. It is observed that the oblique perforation has promising effects in enhancing the unidirectional and/or omnidirectional bandgap efficiency, depending on the topology and perforation angle of OPhP.

## 1. Introduction

Phononic crystals (PhCrs) are lattice structures that can manipulate elastic waves in an extraordinarily way through their periodic microstructure [1,2,3,4,5,6,7,8,9,10]. The main characteristic of PhCrs is the existence of frequency bands (so called bandgaps), over which propagation of an incident wave is banned. Moreover, the wave may be resonated and/or guided inside an intentionally introduced defect in a PhCr, at a frequency in the bandgap frequency range [11,12]. A wider bandgap enables phononic controllability over a wider frequency range, while a lower bandgap frequency range implies that a larger incident wavelength can be manipulated by a specified phononic lattice. Therefore, relative bandgap width (RBW), which is defined as bandgap width divided by mid gap frequency, is normally used to indicate the bandgap efficiency of PhCrs. Furthermore, due to the strong anisotropy that is introduced by PhCrs, they possess flat and concave wave fronts at particular frequencies that can, respectively, be used for self-collimation and focusing of elastic waves [8,13,14].

The bandgap frequency of a PhCr depends on the dimensions, the constitutive material(s), and the topology of its irreducible periodic feature (unit-cell). Phononic crystal plates (PhPs) have promising application in manipulation of guided waves for designing low loss acoustic devices (resonators, filter, and wave guides) [3,15,16,17] and for structural health monitoring purposes [12,18]. PhPs may be produced by periodic placement of stiff inclusions inside a compliant base plate [3,19], by periodic through perforation of a uniform plate [5,20,21,22,23,24], or by attaching a periodic array of pillars on a substrate [15,25], or a combination of them [26]. Perforated PhPs are relatively easy to produce, in which porosities introduce strong reflecting boundaries (i.e., high acoustic impedance mismatch) that are free from interfacial imperfections. Moreover, finite thickness and light weight cellular design of perforated PhPs make them a promising constitutive structural material with low vibration transmission and acoustic radiation. The topology of perforation profile can be optimized such that maximized RBW of a particular mode type (symmetric or asymmetric) or a complete bandgap of mixed guided waves (combination of symmetric and asymmetric modes) is obtained [27,28] and with desired stiffness [5,22,29] or deformation induced tunability [30].

Earlier studies have shown the abnormal dispersion properties of tapered meta-surfaces with axisymmetric non-uniform through the thickness profile [31,32]. In this paper, obliquely perforated PhPs (OPhPs) are introduced for enhanced bandgap efficiency through their non-uniform through-the-thickness cross section. Two distinct designs with symmetric and asymmetric through-the-thickness constitution, with respect to the mid-plane, are proposed. Various unit-cells with arbitrary as well as optimized perforation profile are examined at perforation angles 0° to 60° with respect to the normal axis of the plate. The modal band structure of the first 10 wave modes is calculated and the variation of total RBW with respect to the perforation angle is studied. The results confirm the promising effect of oblique perforation in enhancing partial (unidirectional) and/or complete (omnidirectional) RBW of studied topologies.

The layout of the paper is, as follows. First, the two proposed designs of OPhPs, relevant unit-cells, and selected topologies to be examined are presented and constitutive equations of modal band analysis are given. Then, the calculated modal band structure and RBW of selected topologies with respect to the perforation angle are presented and discussed. Finally, an alternative topology is selected and the enhanced bandgap efficiency of its OPhP is validated through transmission spectrum of its finite phononic structure.

## 2. OPhP Designs and Modal Band Analysis

**Figure 1:**
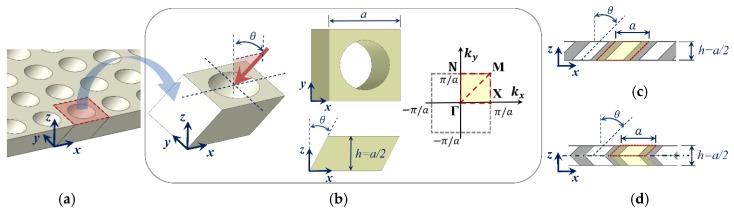
(**a**) Schematic of proposed obliquely perforated porous phononic crystal plates (OPhPs) produced by oblique perforation at an angle θ (about y-axis) on a uniform plate and (**b**) selected unit-cell and relevant irreducible Brillouin zone, and cross section of (**c**) an asymmetric OPhP (A-OPhP) and (**d**) a symmetric OPhP (S-PhP) lattice, respectively, with asymmetric and symmetric through-the-thickness design.

As schematically shown in Figure 1a,b, the proposed OPhP design can be produced by lateral perforation of a uniform background plate at an angle θ with respect to the plate’s normal axis (i.e., z-axis). Figure 1b shows the irreducible unit-cell of OPhP with continuous solid boundary being chosen along the perforation path at an angle θ about y-axis. The perforation profile is assumed to have square symmetry (in xy-plane), and unit-cell with aspect ratio (width to thickness) a/h=2 is considered.

The designs shown in Figure 1c,d introduce asymmetric and symmetric OPhPs, respectively. An asymmetric OPhP (A-OPhP) may be produced by oblique thorough perforation, and a symmetric OPhP (S-OPhP) may be produced by double sided perforation to the mid-plane or by mirrored attachment of two A-OPhPs. The shaded area in Figure 1c,d indicates the border of OPhP unit-cell. In the case of S-OPhP, as shown in Figure 1d, only half of the thickness from mid-plane to the top suffices to be considered as the unit-cell due to its mirrored symmetry. However, appropriate boundary conditions have to be applied to the mid-plane to define this mirrored symmetry [29].

For modal band analysis of the unit-cell, Bloch-Floquet boundary conditions have to be applied to ensure the periodicity of opposing boundaries for the in-plane wave vectors $k=\left\{\begin{array}{cc} k_{x} & k_{y} \end{array}\right\}$ as follows [33]:

(1) $$u\left(x,t\right)=u\left(x+A,t\right)e^{ik\cdot A}$$

where $u={\left\{u\;v\;w\right\}}^{T}$ is the displacement vector corresponding to location vector x={x y z}, A={a a} is the lattice periodicity vector for unit-cell width a, and t is time and $i=\sqrt{-1}$. Contrary to perpendicular perforation, m which leads to a square symmetric unit-cell, oblique perforation reduces the symmetry while the lattice periodicity remains the same in x- and y-axis. Due to the periodicity of boundary condition (Equation (1)), k can be searched over the first Brillouin zone and, according to the common practice, only on its border [34]. The inset of Figure 1b shows the first Brillouin zone in which the irreducible Brillouin zone corresponding to the OPhP unit-cell (confined by the border ΓΧΜΓΝΜ) is highlighted. Obviously, for perpendicular perforation (i.e., θ=0°), which leads to a square symmetric unit-cell, the irreducible Brillouin zone reduces to the triangle ΓΧΜ. By modal analysis of the unit-cell over the $n_{k}$ discrete search points i on the border of the irreducible Brillouin zone, the total RBW within the first 10 modal branches can be initially defined as:

(2) $$\;RBW_{i}\left(\theta \right)=\overset{10}{\underset{j=1}{\sum }}\frac{max\left(min_{i=1}^{n_{k}}\omega_{j+1}^{2}\left(k_{i},\theta \right)-max_{i=1}^{n_{k}}\omega_{j}^{2}\left(k_{i},\theta \right),0\right)}{0.5\;\left(min_{i=1}^{n_{k}}\omega_{j+1}^{2}\left(k_{i},\theta \right)+max_{i=1}^{n_{k}}\omega_{j}^{2}\left(k_{i},\theta \right)\right)}\;$$

where $\omega_{j}\left(k_{i},\theta \right)$ is the modal frequency of mode j at wave vector $k_{i}$ corresponding to point i on the border of the Brillouin zone and at perforation angle θ. RBW is calculated for 13 perforation angles between 0 to 60° with 5° increment. The upper limit of bandgap frequency within the first 10 modal branches varies with perforation angle and it reaches a maximum $\omega_{MB}$ at an angle specific to the topology. Therefore, the RBW of all the perforation angles is consistently recalculated over a constant frequency range of $0<\omega \leq \omega_{MB}$:

(3) $$\;RBW\left(\theta \right)=\overset{20}{\underset{j=1}{\sum }}\frac{max\left(min\left(min_{i=1}^{n_{k}}\omega_{j+1}^{2}\left(k_{i},\theta \right),\omega_{MB}\right)-max_{i=1}^{n_{k}}\omega_{j}^{2}\left(k_{i},\theta \right),0\right)}{0.5\;\left(min_{i=1}^{n_{k}}\omega_{j+1}^{2}\left(k_{i},\theta \right)+max_{i=1}^{n_{k}}\omega_{j}^{2}\left(k_{i},\theta \right)\right)}\;$$

Naturally, sufficiently more modal branches have to be included in Equation (3) (herein 10 more modal branches) to take into account any bandgap emerging below the upper frequency limit $\omega_{MB}$.

The finite element method (FEM) is implemented through ANSYS APDL FEM solver (ANSYS, Inc., Canonsburg, Pennsylvania, USA, Academic Research, Release 16) for modal band analysis of OPhPs. Aluminum with elastic modulus $E_{s}=70\;\mathrm{GPa}$, Poisson’s ratio $\nu_{s}=0.34$, and density $\rho_{s}=2700\;\mathrm{kg}/m^{3}$ is considered as the constitutive material. However, the constitutive material properties do not significantly affect the RBW calculated for the porous design of OPhPs. Two identical unit-cell models are meshed and superimposed by constraint equations, as explained in [29], which one model accounts for the real terms and the other one accounts for the imaginary terms of the periodic boundary condition, as defined in Equation (1). Opposite faces of the unit-cell are modelled by conforming meshes (i.e., each boundary node has a mirrored counterpart on the opposite boundary) to ensure a proper definition of periodicity.

As mentioned earlier, in the case of S-OPhPs, only half of the thickness from mid-plane to the top surface is modelled as the unit-cell. Then, appropriate boundary conditions are applied to the mid-plane to calculate the modal band structure of symmetric and asymmetric guided wave modes individually [29]. However, for A-OPhPs the modal band structure of mixed guided waves is only calculated because its modes with dominant symmetric or asymmetric character cannot be easily decoupled by such boundary condition.

**Figure 2:**
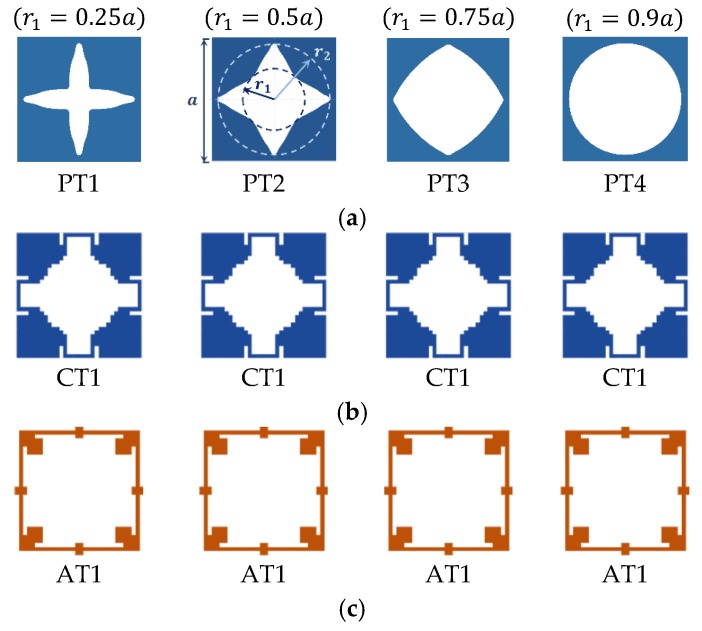
Perforation profiles chosen to study the bandgap efficiency of OPhPs, (**a**) prescribed topologies with arbitrary perforation profile ($r_{2}=0.9a$ and different $r_{1}$), and optimized topologies with (**b**) maximized complete bandgap of mixed guided waves and (**c**) maximized bandgap of asymmetric guided wave modes [10].

In order to demonstrate the bandgap efficiency of OPhPs, a set of perforation profiles with arbitrary and optimized topologies are chosen, as depicted in Figure 2.

An arbitrary perforation profile with square symmetry is prescribed with smoothly varying perforation radius from an inner radius $r_{1}$ to an outer radius $r_{2}$, as shown in Figure 2a. Topologies PT1 to PT4 all have outer radius of 0.9a but various inner radiuses of 0.25a, 0.5a, 0.75a, and 0.9a, respectively, where a is the unit-cell width. Moreover, optimized topologies with maximized RBW of complete bandgap of mixed guided wave modes CT1-CT4 as well as optimized topologies with maximized RBW of asymmetric guided wave modes AT1-AT4 are studied [10]. The topologies were optimized through a multi-objective optimization algorithm for both maximized RBW of fundamental modes and maximized in-plane stiffness for unit-cell with aspect ratio 2. The bandgap efficiency of the selected optimized topologies reduces, while their stiffness increases from CT1 to CT4 and likewise from AT1 to AT4. When oblique perforation shows an increase in a bandgap type (symmetric, asymmetric, or mixed modes) of arbitrary profiles, it is interesting to also examine its efficiency for optimized perforation profiles of the same bandgap type. However, it is not meant that the topologies optimized for perpendicular perforation are still optimized in the case of oblique perforation.

## 3. Bandgap of Mixed Guided Wave Modes by A-OPhPs

**Figure 3:**
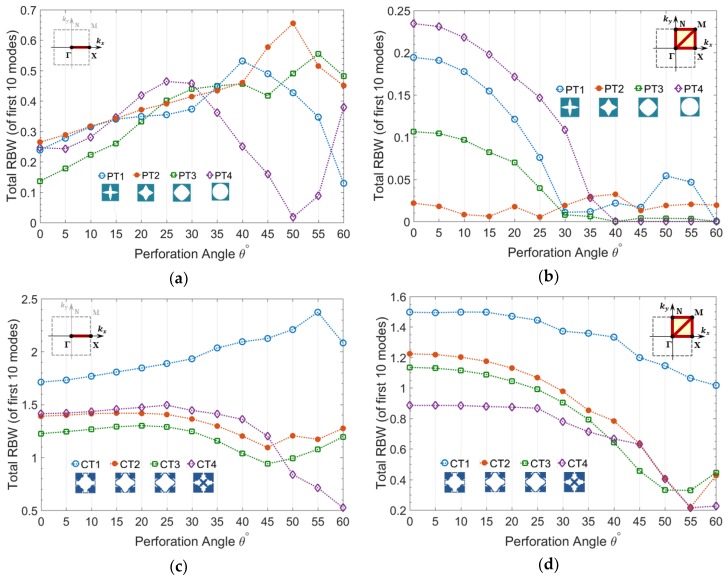
Total relative bandgap width (RBW) of mixed guided wave modes versus perforation angle calculated for (**a**,**b**) prescribed topologies PT1-PT4 and (**c**,**d**) optimized topologies CT1-CT4, (**a**,**c**) partial bandgap in ΓΧ and (**b**,**d**) complete bandgap in ΓΧΜΓΝΜ.

In this section the variation of RBW of A-OPhPs with respect to the perforation angle is presented. The total RBW of mixed guided wave modes is calculated for prescribed topologies PT1-PT4, as well as optimized topologies CT1-CT4 for 13 perforation angles between 0° to 60° with 5° increments, as shown in Figure 3.

According to Figure 3a, the partial bandgap of all the prescribed topologies PT1-PT4, along Brillouin zone border ΓΧ, initially increases by perforation angle and declines after a particular angle depending on the topology of perforation profile.

This partial bandgap corresponds to the unidirectional wave propagation in x-axis along which oblique perforation is performed in zx-plane. Those topologies with lower initial RBW (at θ=0°) reach their first peak at a smaller angle and PT1 with highest initial RBW keeps rising up to the angle θ=50°. Likewise, as demonstrated by Figure 3c, the partial bandgap of all the optimized topologies show an initial increase by perforation angle among which CT1 with highest RBW keeps increasing up to the angle θ=55°. The topologies with lowest initial RBW e.g., PT4, CT2, and CT3, which reach their initial peak at smaller angle, start rising up again after reaching a minimum value. As expected, the RBW of optimized topologies is significantly higher than that of arbitrarily prescribed topologies.

On the contrary, the complete bandgap of all topologies PT1-PT4 and CT1-CT4 do not improve by perforation angle. Figure 3b demonstrates the steep decline of RBW for PT1-PT3 and minor fluctuations of PT4 with lowest initial RBW versus perforation angle. The rate of reduction of RBW versus perforation angle is lower in optimized topologies when compared to prescribed topologies, which are correlated to their initially much higher RBW.

**Figure 4:**
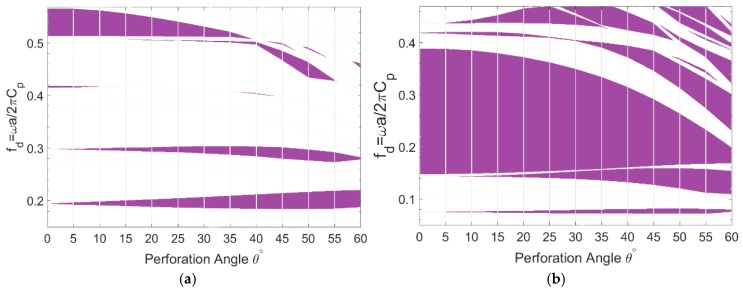
Variation of frequency ranges corresponding to the bandgaps of mixed guided wave modes opened within the first 10 modal branches versus perforation angle 0°≤θ≤60°, (**a**) partial bandgap of prescribed topology PT2 and (**b**) partial bandgap of optimized topology CT1 in ΓΧ.

The frequency ranges corresponding to the partial bandgaps of the first 10 modal branches and their variation with respect to the perforation angle are shown in Figure 4a,b for topologies PT2 and CT1, respectively. Whereas, the actual bandgap frequency of the unit-cell depends on its periodicity a and constitutive material properties, it is common practice to calculate a dimensionless frequency $f_{d}=\omega a/2\pi C_{p}$ where $C_{p}=\sqrt{E_{s}/\rho_{s}}$.

As demonstrated by Figure 4a concerning the topology PT2, two fundamental partial bandgaps, just below $f_{d}=0.2$ and around $f_{d}=0.3$ open, widen, and develop towards lower frequency ranges by perforation angle, which have the most contribution in increased RBW of A-OPhP. Another gap opens above $f_{d}=0.5$ from perforation angle 15°, widens towards lower frequencies, and closes at angle 55°. However, the highest bandgap narrows down by perforation angle and closes at 40°. Other minor gaps are also present, particularly at larger angles.

Likewise, the partial bandgaps of the topology CT1, as shown in Figure 4b are deviated towards lower frequencies and new bandgaps are introduced as the perforation angle increases. The maximized bandgap of CT1 is gradually narrowed down by perforation angle, however, two fundamental bandgaps emerge and widen at lower frequencies: one from 5° and the other from 10°.

From Figure 4, it is obvious that the gradient of total RBW with respect to the perforation angle strongly depends on the number of modal branches included in calculation of RBW. However, common observations from partial bandgaps of both prescribed topology PT2 and optimized topology CT1 are:

- increasing perforation angle introduces and/or widens fundamental low frequency bandgaps;
- increasing perforation angle shifts higher order bandgaps towards lower frequency levels; and,
- narrowing of a bandgap is generally associated with development of a lower bandgap.

Obviously, all the above observed behavior contribute to the increasing of RBW with perforation angle.

## 4. Bandgap of Asymmetric Wave Modes by S-OPhPs

In this section, the variation of RBW of S-OPhPs with respect to the perforation angle is presented. As discussed in Section 2, half of S-OPhP’s thickness from mid-plane is modelled and symmetric and asymmetric guided wave modes are decoupled by applying appropriate boundary condition to the mid-plane. Partial and complete bandgaps of both wave mode types are calculated for prescribed topologies PT1-PT4, as well as optimized topologies AT1-AT4 for the 13 perforation angles between 0 to 60° with 5° increments.

**Figure 5:**
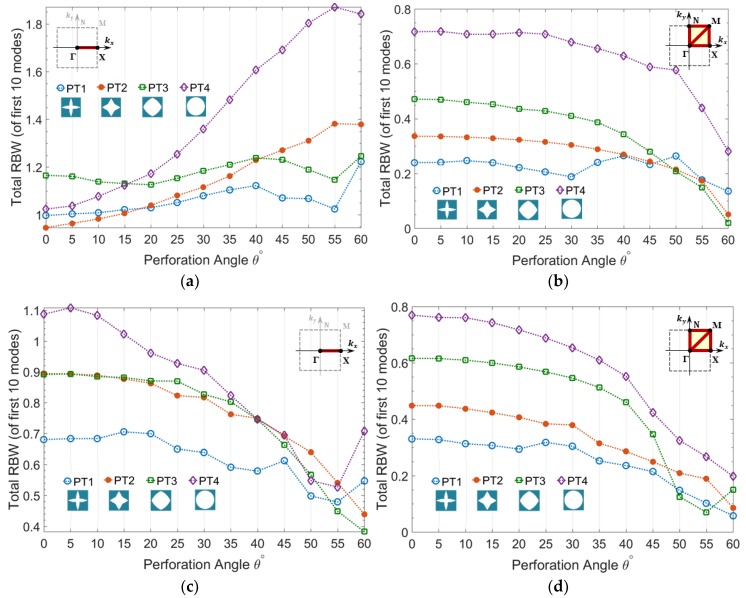
Total RBW of guided wave modes versus perforation angle calculated for prescribed topologies PT1-PT4, (**a**,**b**) asymmetric wave modes and (**c**,**d**) symmetric wave modes, (**a**,**c**) partial bandgap in ΓΧ, and (**b**,**d**) complete bandgap in ΓΧΜΓΝΜ.

The results concerning prescribed topologies PT1-PT4 for both asymmetric and symmetric wave modes are shown in Figure 5 to compare their sensitivity to oblique perforation. According to Figure 5a, the partial RBW of asymmetric wave modes in Brillouin zone border ΓΧ is increased by perforation angle for all prescribed topologies, among which PT4 shows the highest increase rate.

Regarding the complete bandgap of asymmetric wave modes, as shown in Figure 5b, the topology PT1 shows a minor increase of RBW at 40° and 50°, while the RBW of other topologies constantly decline by perforation angle. No improvement is observed in partial and complete bandgap of symmetric modes by perforation angle, as demonstrated in Figure 5c,d. It is noteworthy that RBW of mixed guided waves was also calculated (not shown) and its was observed that the bandgaps degrade by perforation angle.

**Figure 6:**
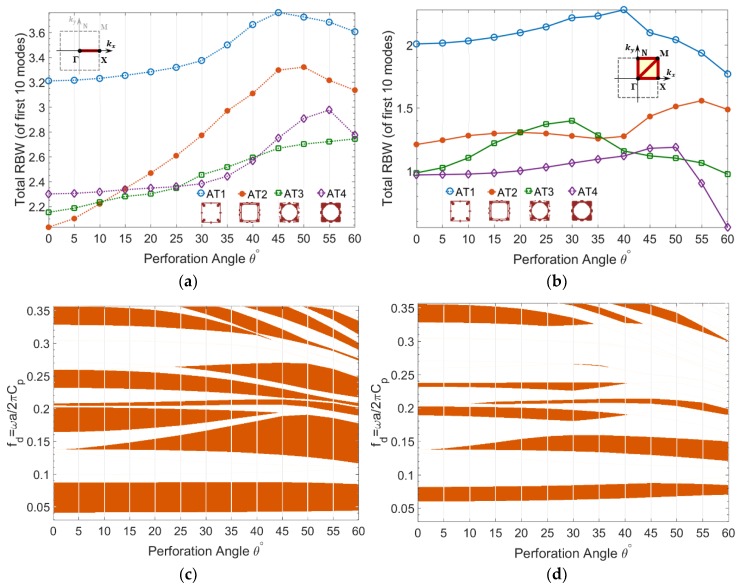
(**a**,**b**) Total RBW of asymmetric guided wave modes versus perforation angle calculated for optimized topologies AT1-AT4, and (**c**,**d**) corresponding bandgap frequency ranges for topology AT3, (**a**,**c**) partial bandgap in ΓΧ and (**b**,**d**) complete bandgap in ΓΧΜΓΝΜ.

Furthermore, RBW of asymmetric wave modes are calculated for optimized topologies AT1-AT4 having maximized bandgap of fundamental asymmetric wave modes, as shown in Figure 6. The results confirm that both partial and complete bandgaps of asymmetric wave modes are enhanced by perforation angle in the S-OPhP design of all optimized topologies AT1-AT4. The sensitivity of RBW to the perforation angle strongly depends on the topology. For example, both topologies AT3 and AT4 have almost the same initial complete RBW (Figure 6b). However, AT3 shows higher increase rate up to the angle 30° and AT4 shows a considerably lower increase rate up to a larger perforation angle 50°.

The gradient of frequency ranges corresponding to the partial and complete bandgaps of optimized topology AT3 with respect to the perforation angle is also demonstrated in Figure 6. From Figure 6c, it is evident that the lowest partial bandgap is almost insensitive to the perforation angle. A second partial bandgap emerges at around $f_{d}=0.14$ below 5° and it keeps widening and inclining towards lower frequency ranges by the perforation angle, while the third bandgap above it keeps narrowing down by the perforation angle.

This trend of bandgap narrowing is generally associated with the emergence and/or widening of another bandgap between lower modes, and as such, leads to increasing RBW. The same trend is also observed for complete bandgaps of AT3, as demonstrated in Figure 6d. The lowest bandgap, which is the maximized fundamental bandgap of topology, slightly shifts towards higher frequencies by perforation angle and a second bandgap emerges at around $f_{d}=0.14$ below 5° and widens and inclines towards lower frequencies.

**Figure 7:**
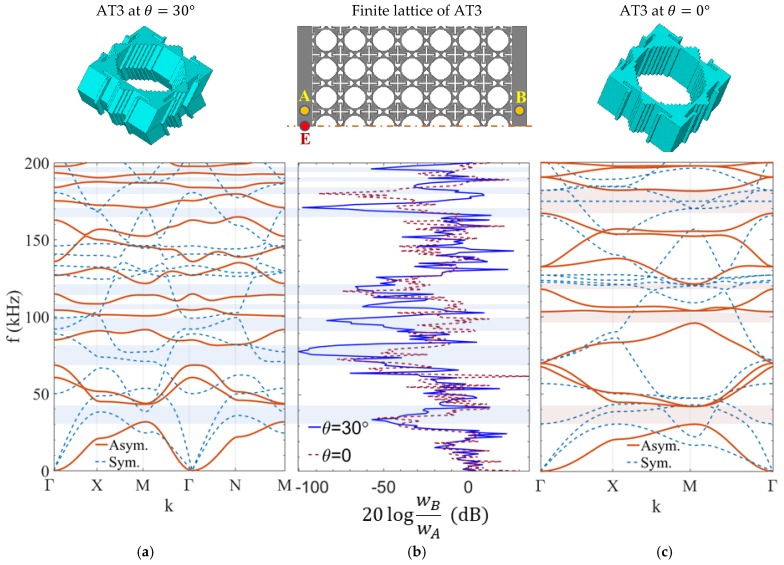
Modal band structure of S-OPhP of topology AT3 at perforation angles (**a**) 30° and (**c**) 0°, and (**b**) transmission spectrum in a finite phononic structure for both perforation angles.

Furthermore, the bandgap efficiency of S-OPhPs is validated by calculating the transmission spectrum of a finite phononic structure of topology AT3 for perforation angles 0° and 30° (Figure 7). The modal band structure of both perforation angles is calculated for an aluminum unit-cell of size a=10 mm and thickness h=5 mm, as demonstrated in Figure 7a,c. The symmetric model of a square phononic array of 7×7 unit-cells, as shown on top of Figure 7b is modeled and is subjected to an out-of-plane harmonic excitation $w_{E}=1\;\mu m\;$on the top surface at point E. This approach leads to dominant excitation of asymmetric wave modes. The transmission spectrum of the out-of-plane displacement from an arbitrary point A on the excitation side to an arbitrary point B on the other side of the phononic structure is then calculated as $20\log\frac{w_{B}}{w_{A}}\;\mathrm{dB}$, as depicted in Figure 7b. 

The dips of the transmission spectrum correspond to the phononic bandgap frequencies over which the amplitude of elastic waves is highly attenuated. The calculated modal band structures of the unit-cells are in excellent agreement with the transmission spectrum of this finite phononic structure, and thus confirm the enhancement of the complete RBW of S-OPhPs in a finite structure. The transmission spectrum of the lowest (i.e., optimized) bandgap of AT3 shows slightly higher attenuation at perforation angle 30°, which may be due to the contribution of symmetric wave modes in the transmission spectrum of perforation angle 0°. In contrast to the unchanged bandgap efficiency of the fundamental bandgap, multiple extra dips are present at higher frequency in the transmission spectrum of perforation angle 30° when compared to the transmission spectrum of perforation angle 0°. 

## 5. Conclusions

Obliquely perforated phononic crystal plates were introduced in this paper with a symmetric (S-OPhP) and an asymmetric (A-OPhP) design. Modal band structure of various oblique perforation angles were calculated and the sensitivity of total RBW of the first 10 modal branches with respect to the perforation angle was investigated. Perforation profiles for arbitrary unit-cell topologies, as well as optimized unit-cell topologies (with maximized RBW of fundamental modes), were examined.

Partial (unidirectional) bandgaps along the plane of perforation angle as well as complete (omnidirectional) bandgaps were evaluated. It was observed that for an A-OPhP design, the partial RBW of mixed guided wave modes increases by perforation angle for all of the selected topologies, and it reaches a maximum at a perforation angle, which is specific to the topology. Moreover, by a S-OPhP design the partial bandgap of asymmetric wave modes increases for all of the selected topologies, and likewise reaches a maximum at a perforation angle specific to the topology. S-OPhP design also proved to enhance the complete bandgap of those topologies with maximized bandgap of asymmetric wave modes.

A general trend was observed that narrowing of a bandgap by perforation angle is normally associated with emergence and/or widening of another bandgap between lower modes, which leads to an increasing RBW by perforation angle.

It is concluded that introducing a constant perforation angle throughout the phononic lattice can significantly enhance its unidirectional and/or omnidirectional bandgap efficiency, depending on the topology of perforation profile. This fact inspires the idea of simultaneous optimization of perforation profile and perforation angle. Varying the azimuth angle of perforation over the perimeter of the perforation profile introduces conical like cavities, which may lead to supreme omnidirectional bandgap efficiency.
